# Diagnosis of *mycobacterium avium* complex infection utilizing metagenomics next-generation sequencing: a case report

**DOI:** 10.3389/fmed.2023.1247034

**Published:** 2023-10-18

**Authors:** Hongli Li, Luqing Wei, Fenge Li

**Affiliations:** ^1^Department of Respiratory, Tianjin Beichen Hospital, Tianjin, China; ^2^Core Laboratory, Tianjin Beichen Hospital, Tianjin, China

**Keywords:** nontuberculous mycobacterium, *Mycobacterium avium*, pulmonary disease, metagenomics next-generation sequencing, diagnosis

## Abstract

*Mycobacterium avium-intracellulare* complex (MAC) is a type of nontuberculous mycobacteria (NTM) and is associated with underlying pulmonary diseases, such as chronic obstructive pulmonary disease, bronchiectasis, chronic aspiration or recurrent pneumonia, inactive or active tuberculosis, pneumoconiosis, and bronchogenic carcinoma. The risk factors for NTM-PD include host, drug, and environmental factors. In this report, we present the case of a 61-year-old man who developed bilateral lung nodules and was experiencing severe hemoptysis. The repeat acid-fast bacilli test performed on both sputum and bronchoalveolar lavage fluid (BALF) samples showed a negative result, as did the GeneXpert test. We employed metagenomic next-generation sequencing (mNGS) to analyze the lung nodule and BALF samples collected from the patient. Both samples tested positive for MAC within 3 days. In addition, traditional MAC culture, conducted for 2 months, confirmed the growth of MAC in the patient’s BALF. Then, the patient was treated accordingly. Following treatment, a high-resolution chest computed tomography scan revealed a significant reduction in lung nodules of the patient after 2 months. These results indicate that MAC-associated lung nodules were responsible for the patient’s symptoms, emphasizing the need for vigilance in diagnosing MAC infection in the patient without predisposing conditions. Furthermore, these results highlight the potential utility of mNGS as a promising rapid diagnostic tool for MAC infection and its potential role in the diagnosis of NTM disease.

## Introduction

*Nontuberculous mycobacteria* (NTM) refer to species of mycobacterium that do not belong to *Mycobacterium tuberculosis* complex or *Mycobacterium leprae*. NTM are known to cause both pulmonary and extrapulmonary illnesses in humans of all ages (1), with *Mycobacterium avium* complex (MAC) being the most common type. MAC consists of a growing number of species, including *M. arosiense*, *M. bouchedurbonense*, *M. timonense*, *M. vulneris*, and *M. yongonense*. NTM-related pulmonary disease (NTM-PD) is a common type of NTM infection (2–5). Predisposing conditions such as chronic obstructive pulmonary disease, bronchiectasis, chronic aspiration or recurrent pneumonia, inactive or active tuberculosis, pneumoconiosis, and bronchogenic carcinoma are present in 54–77% of the patients with pulmonary MAC disease. Risk factors for NTM-PD include host, drug, and environmental factors (6). Few studies have investigated the value of metagenomic next-generation sequencing (mNGS) for diagnosing MAC infection. Here, we report a case of a man with pulmonary disease caused by MAC that was promptly and effectively diagnosed using metagenomic next-generation sequencing (mNGS), leading to a timely and precise treatment for the patient following the diagnosis.

## Case presentation

**Figure 1:**
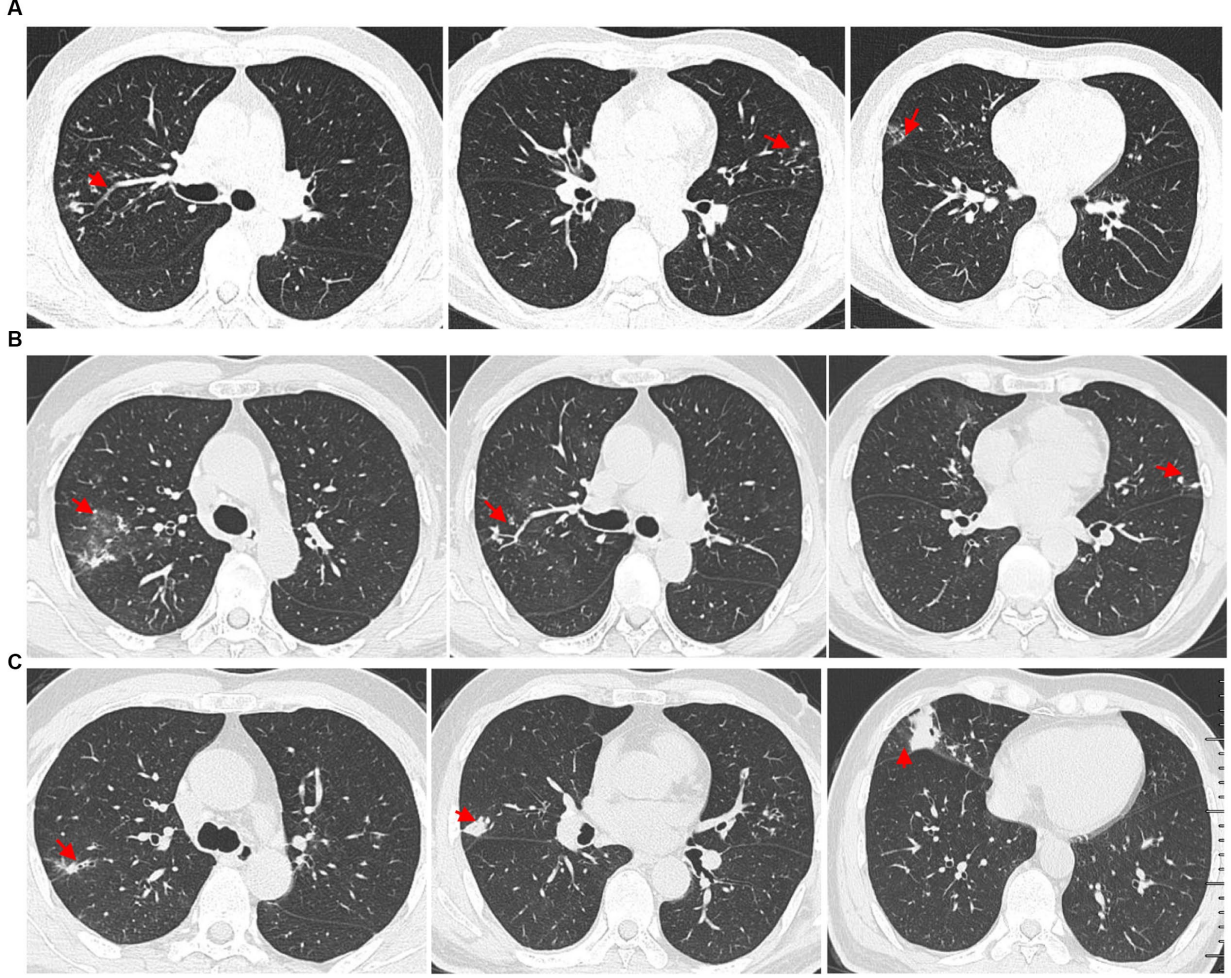
High-resolution chest computed tomography (CT) scan results from different periods. **(A)** CT scan results from different layers taken on 14 January 2019. The multiple small nodules (red arrow) in the right upper lobe, left upper lobe, and right middle lobe. **(B)** The patient underwent a CT scan of the chest on 7 July 2021, which showed ground glass opacity in the right upper lobe (red arrow) and multiple small nodules in both upper lobes (red arrow). **(C)** Outpatient reexaminations using a repeat CT scan on 19 August 2021 revealed an increase in the size of multiple small nodules in the upper and middle lobes (red arrow).

A 61-year-old man presented with bilateral lung nodules in chest roentgenograms (Figure 1A) during his routine health examination in January 2019. The bilateral lung nodules remained stable during subsequent regular outpatient reexaminations in February 2019, December 2019, and December 2020. Particularly, this patient showed no clinical symptoms throughout this period. However, in July 2021, the patient was admitted to our hospital due to hemoptysis. He did not display symptoms commonly associated with infection, such as fever, dyspnea, pyrosis, night sweats, or weight loss at that time. Additionally, the physical examination results were also normal. All laboratory tests of the patient, including peripheral blood count, renal and liver functions, indirect immunofluorescence test for antinuclear (ANA) antibodies, rheumatoid factor test, anti-cyclic citrullinated peptide test, anti-extractable nuclear antigen test, lymphocyte subtype test, urine test, and stool test showed normal results. The patient underwent a chest computed tomography (CT) scan, revealing multiple small nodules in both upper lobes and ground glass opacity in the right upper lobe (Figure 1B). The nodules were found to be similar in size to the ones found in the original CT scan from January 2019, but ground glass opacity was observed. Subsequently, the patient underwent bronchoscopy examination, and bronchoalveolar lavage fluid (BALF) samples were collected for further pathogen analysis. The results of the acid-fast bacilli (AFB) test and GeneXpert MTB/RIF test for sputum and BALF were all negative. The patient then received antibiotics treatment with moxifloxacin, while he did not show hemoptysis during his stay at the hospital. The patient did not show any symptoms throughout the 5-day course of antibiotics treatment and was subsequently discharged from the hospital.

**Figure 2:**
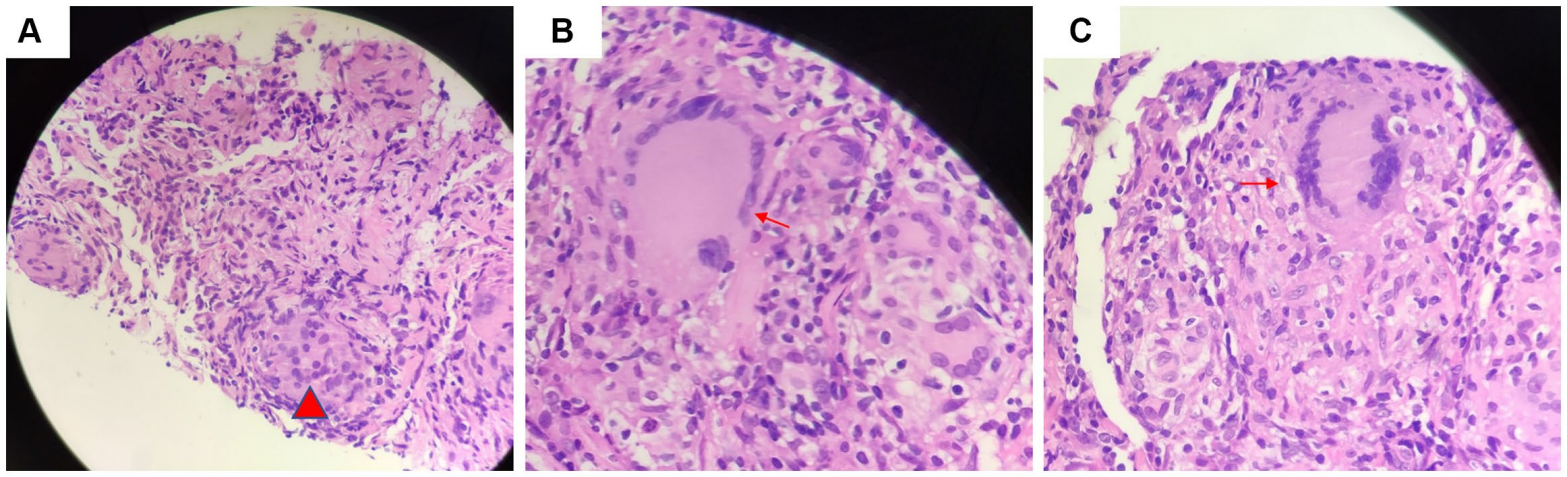
Tissue acid-fast staining indicated the presence of **(A)** granulomatous lesions (red arrow) and **(B)** scattered giant multinucleated giant cell (red arrow). **(C)** There was no caseous necrosis observed (red arrow).

**Figure 3:**
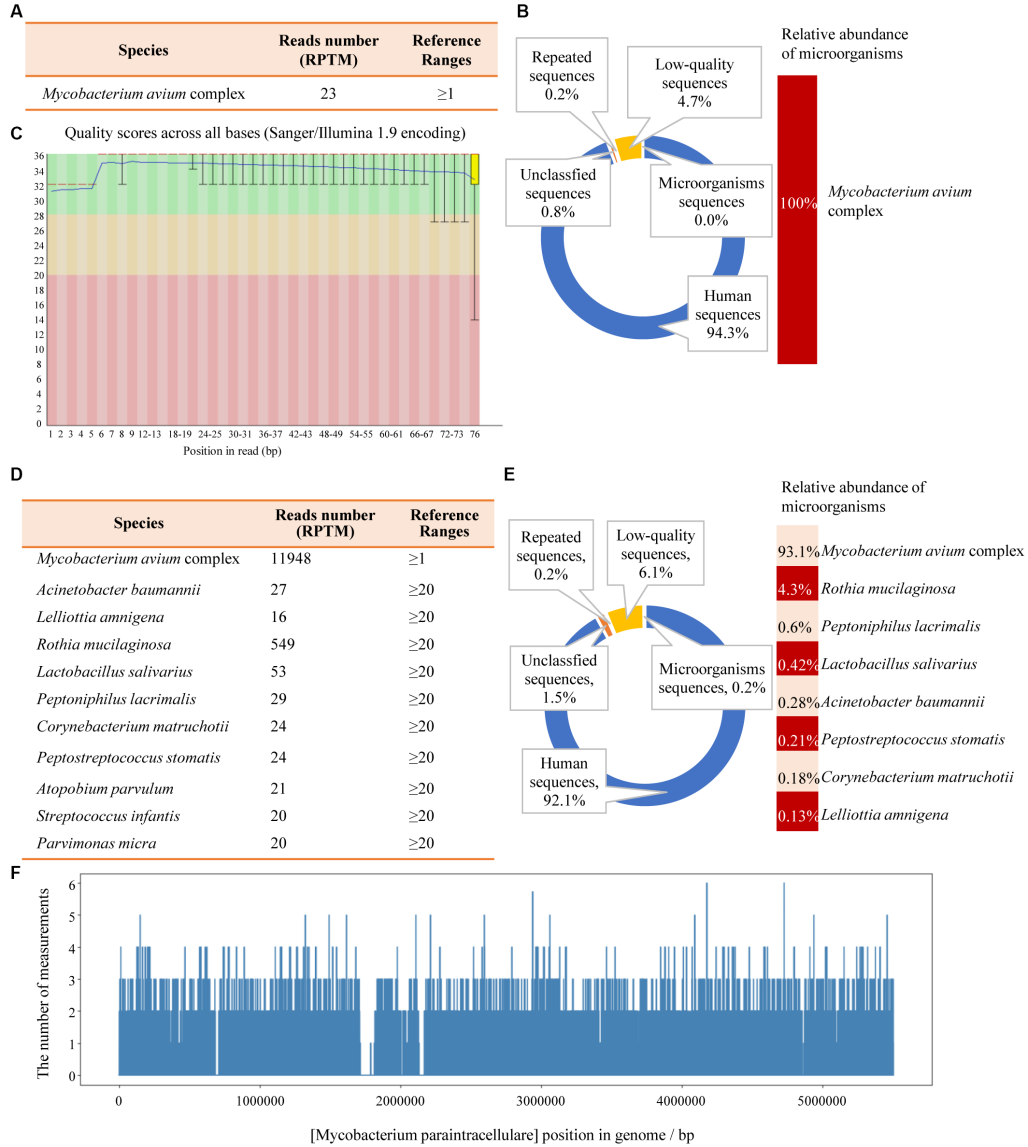
The mNGS results of the patient’s tissue and BALF. **(A)** The identified pathogen in the tissue was *Mycobacterium avium* complex (MAC). **(B)** The relative abundance of MAC accounts for 100% of the identified microorganisms in tissue. **(C)** The results of single base quality distribution in the tissue sample. **(D)** MAC was also identified in the BALF sample. **(E)** 93.1% of the reads of microorganisms were identified in BALF that belongs to MAC. **(F)** Coverage of MAC detected by BALF mNGS.

**Figure 4:**
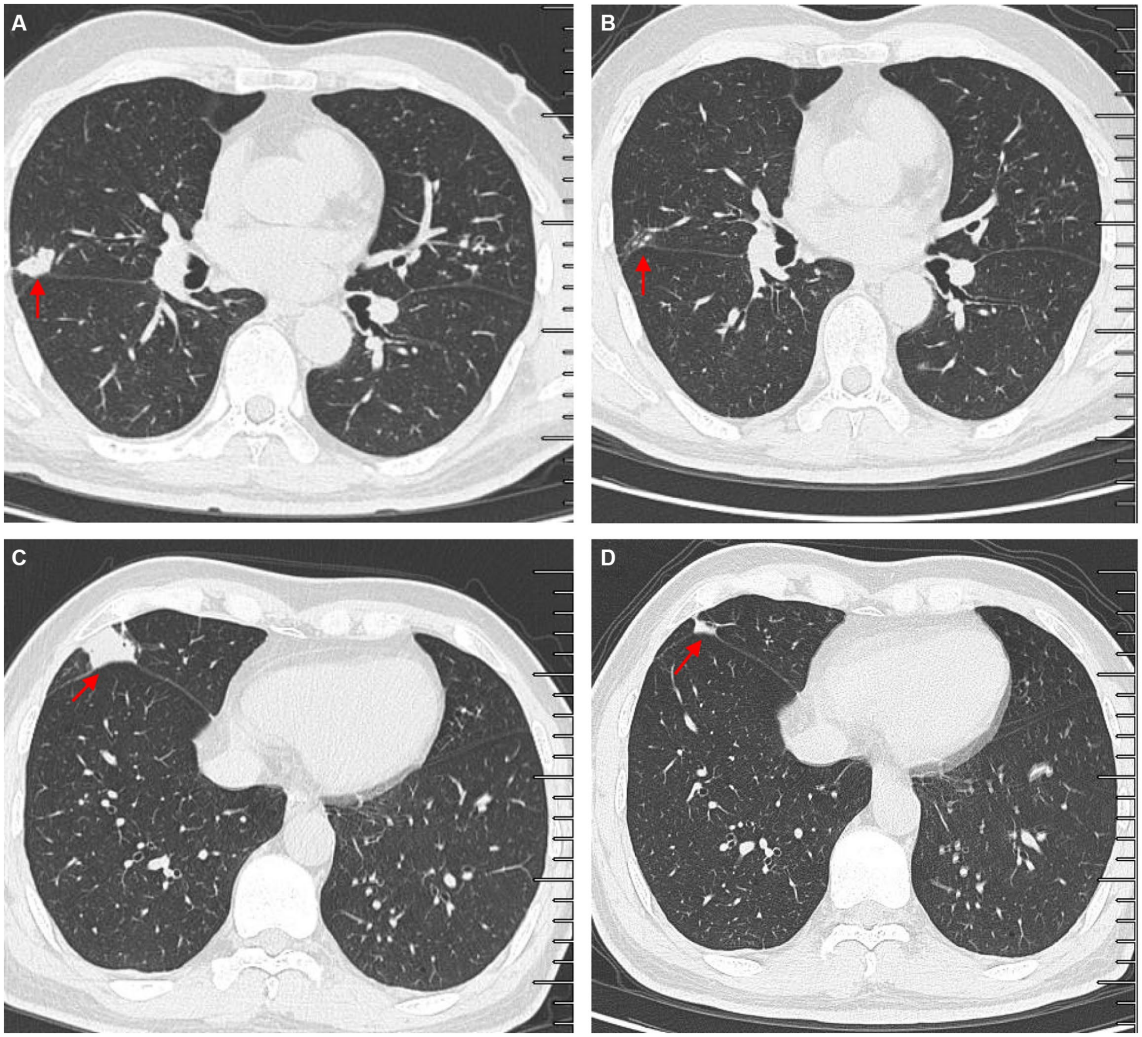
Comparisons of outpatient reexaminations of chest computed tomography (CT) scan on 19 August 2021 and 21 October 2021. The results showed that the nodules in the middle lobes observed on 21 October 2021 **(A,C)** became much smaller than those observed on 19 August 2021 **(B,D)**. Red arrow–lung nodules.

Another outpatient reexamination on 19 August 2021 using a repeat high-resolution chest CT scan revealed a noticeable increase in the size of the multiple small nodules in the upper and middle lobes of the lung (red arrow) (Figure 1C). A chest CT-guided lung biopsy was performed. The histopathology (right lung biopsy) showed the alveolar tissue, local lymphocyte infiltration, granulomatous lesions, scattered Langerhans giant cells, some fibrinoid exudates along with no caseous necrosis. The result of the tissue acid-fast staining was negative (Figure 2). As such, a second bronchoscopy was performed, and BALF was collected for pathogen testing. However, both acid-fast bacilli test and GeneXpert MTB/RIF test results for BALF were negative. Particularly, this patient still experienced no clinical symptoms of infection during this time. On 24 August 2021, the lung tissue and BALF of the patient were subjected to mNGS testing, and 23 reads (Figures 3A,B) and 11,948 reads (Figures 3D–F) of MAC were identified within 3 days. Moreover, the MAC infection was confirmed again through a BALF culture test conducted 20 days later. The patient was then treated with a combination of ethambutol (25 mg/kg three times per week), rifampicin (600 mg three times per week), and azithromycin (500 mg three times per week) after breakfast for 6 months. Notably, the patient did not experience any additional symptoms during these drug treatments. It is also worth noting that no drug-related adverse reactions were observed throughout the treatment period. Moreover, a significant decrease in nodule size was observed in a high-resolution chest CT scan which was performed 2 months after the aforementioned combination therapy (Figure 4).

## Method of metagenomic next-generation sequencing

Bronchoalveolar lavage fluid (BALF, 3–5 mL) was collected from patients in sterile tubes with anticoagulant, following standard procedures. The samples were then transported at hypothermia at 4–8°C after collection. Subsequently, DNA extraction was performed using PathoXtract^®^ Basic Pathogen Nucleic Acid Kit (WYXM03211S, WillingMedCorp, Beijing, China), following the manufacturer’s protocol. Then, DNA libraries were constructed using the Illumina^®^ DNA Prep, (M) Tagmentation kit (20018705, Illumina) according to the manufacturer’s protocol. DNA libraries were sequenced on NextSeq^™^ 550Dx system using a 75 bp, single-end sequencing kit (Illumina), and at least 20 million sequencing reads were acquired for each sample. A negative control sample (nuclease-free water) was processed and sequenced in parallel in each sequencing run for quality control. Finally, the raw FASTQ-format data were subjected to Trimmomatic v0.40 for quality control and evaluation, whereby low-quality or undetected sequences, sequences contaminated by splices, high-coverage repeats, and short-read-length sequences were filtered out. High-quality sequencing data were compared with the human reference genome GRCh37 (hg19) using Bowtie2 v2.4.3, enabling the removal of human host sequences. Using Kraken2 v2.1.0 software, the remaining sequences were aligned to four databases downloaded from NCBI, including bacteria, fungi, viruses, and parasites, for classification. For identification of the pathogens, a RPTM value, which defined as a detected number of pathogen specific reads per 10 million was used. RPTM ≥3 was used as an empirical threshold for virus detection. For bacteria and fungi, positive pathogens were required to meet a RPTM threshold of ≥20. Special pathogens (including *Cryptococcus* and *Mycobacterium*) with RPTM ≥1 was identified as positive.

## Discussion

MAC is the most common microbe for nontuberculous mycobacterial associated pulmonary disease (2–5). It contains two major microbe species, *Mycobacterium avium* and *Mycobacterium intracellulare* (7). MAC are commonly isolated from water, house dust, and soil. Patients become infected with MAC primarily through inhalation, ingestion, invasive procedures, or trauma (8). Host-environment interactions are the main cause of MAC-PD (9), and the following are risk factors that contribute to the development of MAC-PD: (1) patients with smoking and alcoholism history (8, 10); (2) elderly white men with underlying pulmonary diseases, such as active tuberculosis, bronchiectasis, pneumoconiosis, chronic aspiration pneumonia, lung cancer, and chronic obstructive pulmonary disease (COPD) (11); (3) non-smoking women over the age of 50 with a normal immune system and no underlying lung disease (12); (4) cystic fibrosis carriers (13); and (5) patients with immunosuppression, such as acquired immunodeficiency syndrome (AIDS) (12–14). Clinical symptoms of MAC-PD, including chronic cough, expectoration, fatigue, dyspnea, hemoptysis, and chest pain, are non-specific. Patients with MAC-PD less frequently exhibit fever and weight loss than patients with typical tuberculosis (12). It has been reported recently that a 65-year-old man with NTM presented with recurrent fever and cough, and the CT scan of the chest revealed a lung infection. The patient’s clinical condition did not improve because they received an incorrect diagnosis and treatment previously. With the help of mNGS, the pathogen was identified as MAC. Subsequently, he received accurate antibiotics treatment, resulting in significant improvements in clinical symptoms and CT scan performance (15). Further, Liu et al. (16) have also reported the effectiveness of mNGS in facilitating and improving the clinical diagnosis of NTM infections.

In this case report, we present the case of a man with a smoking history and no underlying lung-related diseases who experienced only a short-term hemoptysis. The patient’s MAC-PD infection was diagnosed using mNGS technology in only 3 days, which emphasizes the importance of rapid and accurate recognition of MAC infection for timely initiation of effective treatment and symptom alleviation. Therefore, mNGS diagnosis can be precise and rapid when compared to conventional bacteria culture testing. It is worth noting that the patient’s MAC infection was diagnosed rapidly using mNGS, as the patient transitioned from asymptomatic to symptomatic. This led to successful treatment for the patient. This case study can provide valuable clinical experiences for the doctors in the field. Further, the clinical symptoms of this patient were mild and minimal with increasing lung nodules, which was very different from the cases reported previously (15, 16), indicating that clinical symptoms may not be easily noticeable in healthy populations infected with MAC.

Most researchers contend that the histopathology of MAC-PD is comparable to that of TB. Key features of pathology of MAC-PD include (1) the presence of necrotizing granulomas characterized by a central zone of necrosis surrounded by a variably thick rim of epithelioid histiocytes, including pink (“caseating”) necrosis, dirty necrosis (basophilic, rich in nuclear debris), and suppurative necrosis; (2) granulomatous rims that may contain Langhans-type giant cells that are multinucleated; and (3) the presence of atypical lesions with tissue cell aggregation granulomas that are absent or poorly formed, which is normally described as a characteristic reaction in patients with AIDS. Because of these similarities, it is difficult to distinguish TB-and MAC-infected tissue (6, 17, 18). Jing et al. found that pulmonary TB exhibits more pink necrosis and basophilic necrosis, while MAC-PD displays multinucleated giant cells (Figure 4) (19). MAC-PD may also feature suppurative necrosis and vasculitis (non-necrotizing), mimicking granulomatosis with polyangiitis (20). In this study, the pathology test of the patient revealed local lymphocytic infiltration, granulomatous lesions, and scattered Langerhans giant cells, minimal fibrinoid exudates, and no caseous necrosis, and special staining resulted in acid-fast positive. MAC infection manifests as lung nodules in healthy individuals and are characterized by necrotizing granulomatous inflammation containing AFB. However, the pathological manifestations of TB and MAC infection are indistinguishable.

According to the Infectious Disease Society of America and the American Thoracic Society, patients with respiratory symptoms and imagological evidence of pulmonary disease, such as cavitary or nodular opacities on chest radiograph or multifocal bronchiectasis through high-resolution computed tomography, can be diagnosed with fungal infection if malignancy was excluded. Several findings also support the diagnosis of MAC infection: (1) positive culture results from at least two separate expectorated sputum samples and/or NTM molecular positivity for the same pathogen; (2) positive culture results from at least one bronchial wash or lavage and/or NTM molecular positivity for at least one; (3) positive mycobacterial histologic features (granulomatous inflammation or AFB) of transbronchial or lung biopsies, and positive culture for MAC and/or molecular positivity; (4) transbronchial or lung biopsy exhibiting mycobacterial histologic features (granulomatous inflammation or AFB), and one or more sputum or bronchial washing samples that cultured positive for MAC and/or molecular positivity (6). In this study, we determined the diagnosis of MAC-PD based on multiple factors: (1) respiratory symptoms; (2) progression confirmed by high-resolution CT; (3) positive culture result of BALF; (4) histopathological test; (5) mNGS positive results; and (6) effective anti-MAC treatment.

Symptoms of MAC-PD are usually non-specific, and traditional methods for detecting pathogenic microorganism detection have limitations, making it challenging to accurately identify the strains in clinical practice. It is often misdiagnosed as TB or other AFB-positive bacilli. Furthermore, due to lipid-rich outer membranes, MAC are relatively resistant to chemical disinfection by chlorine, chloramines, and ozone, and MAC cultures are usually detected with particular assays. Therefore, positive MAC culture can be detected through direct examination of sputum staining or an acid-fast staining within 7–14 days, and by conventional bacterial culture methods within 21–28 days or even longer. Kodaka et al. (20) found that serum anti-GPL-core IgA testis is useful for diagnosing MAC-PD and determining patient prognosis. While MAC culture serves as the gold standard for diagnosis, it is not routinely tested due to the extended time required for pathogenic NTM to grow, typically taking 2 months. Laboratory tests of p-nitrobenzoic acid selective media culture, polymerase chain reaction (PCR), and MTP64 antigen detection are widely used for identifying TB and NTM (21). In recent years, laboratory testing methods have shifted from biochemical approaches to molecular techniques, and mass spectrometry (MS/MS) has shown promising results for providing rapid speciation of MAC (22, 23). However, the clinical application of MS/MS is limited due to the need for expensive instruments and high technical requirements. The rapid development of mNGS has enabled rapid and specific identification of NTM with high sensitivity (24, 25). In this case report, MAC diagnosis was achieved within 3 days using mNGS.

## Conclusion

The incidence and prevalence of MAC-PD are increasing worldwide and can be a cause of AIDS, bronchiectasis, and immunosuppression. We emphasize the importance of identifying MAC in patients without predisposing conditions. Advancements in diagnostic technology make mNGS a promising diagnostic method for MAC infection and associated diseases in the clinic.

## Ethics statement

The studies involving humans were approved by Ethics committee of Tianjin Beichen Hospital. The studies were conducted in accordance with the local legislation and institutional requirements. The participants provided their written informed consent to participate in this study. Written informed consent was obtained from the individual(s) for the publication of any potentially identifiable images or data included in this article.

## Author contributions

HL, LW, and FL contributed to conception and design of the study and wrote sections of the manuscript. HL organized the database and wrote the first draft of the manuscript. All authors contributed to the article and approved the submitted version.
